# Supplementary material for: Cloud BioLinux: pre-configured and on-demand bioinformatics computing for the genomics community
Source: BMC Bioinformatics. 2012 Mar 19;13:42. doi: 10.1186/1471-2105-13-42 (PMC3372431; doi:10.1186/1471-2105-13-42)
Supplement: Additional file 1 — Supplementary 1 Cloud BioLinux software documentation in the form of a mini, self-contained website. Users need to download and uncompress the .zip file, and open through a web browser the "index.html" file available on the main directory. (ZIP 1823 kb). [file 1471-2105-13-42-S1.ZIP › Cloud-BioLinux-Package-Documentation/docs/dnadist.html]

Bio-Linux Software Documentation Pages

Back to search form

## dnadist

|  |  |
| --- | --- |
| Name | dnadist |
| Description | **dnadist** is part of the PHYLIP package  Copyright 1986-2004 by the University of Washington. Written by Joseph Felsenstein. Permission is granted to copy this document provided that no fee is charged for it and that this copyright notice is not removed.  This program uses nucleotide sequences to compute a distance matrix, under four different models of nucleotide substitution. It can also compute a table of similarity between the nucleotide sequences. The distance for each pair of species estimates the total branch length between the two species, and can be used in the distance matrix programs FITCH, KITSCH or NEIGHBOR. This is an alternative to use of the sequence data itself in the maximum likelihood program DNAML or the parsimony program DNAPARS.  **References:**  Felsenstein, J. 1993. PHYLIP (Phylogeny Inference Package) version 3.5c. Distributed by the author. Department of Genetics, University of Washington, Seattle.    Felsenstein, J. 1989. PHYLIP -- Phylogeny Inference Package (Version 3.2). Cladistics 5: 164-166. |
| Homepage | http://evolution.genetics.washington.edu/phylip.html |
| Remote Documentation | http://evolution.genetics.washington.edu/phylip/doc/dnadist.html |
